# Tri-State Medical Society

**Published:** 1881-10

**Authors:** 


TRI-STATE MEDICAL SOCIETY.—This society, composed of members of the profession in Indiana, Illinois, and Kentucky, will hold its next annual meeting in the city of St. Louis, Missouri, commencing on the morning of the 25th of October, 1881, and continuing three days. We hope our brethren on the west bank of the Mississippi will not be frightened by the invasion of their territory, and that the members of the society after enjoying an unusual "good time," will all succeed in finding their way back to their respective States in safety.

IN view of the opinions lately expressed by eminent oculists, that the reading of German text is injurious to the eyes, the Bernese Government has resolved as much as possible to discourage its use, and all their official announcemeuts and reports will henceforth be printed exclusively in Roman characters.

